# 2015 Terasima Award

**DOI:** 10.1093/jrr/rrv048

**Published:** 2015-07-30


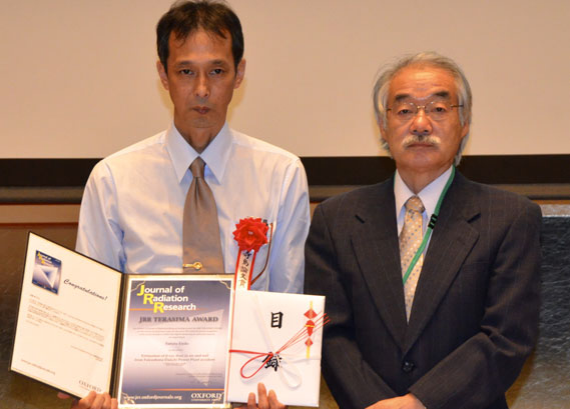


## Comment from the Editor-in-Chief

The Board of Editors of the Journal of Radiation Research is pleased to announce that Dr. Satoru Endo and co-authors are the winners of the 2015 Terasima Award, given for their outstanding paper published in the Journal of Radiation Research in 2013 and 2014. In 2001, the Journal of Radiation Research established the Terasima Award honoring Dr. Toyozo Terasima, a pioneer in Japan in the field of radiation research, and has since been presented to the most prominent paper each year. In 2015, three papers were nominated for the Award and one paper by Dr. S. Endo et al. received the highest evaluation. He and the co-authors received a certificate, a 100,000 yen award, and exemption from processing charges on their next article published in the Journal of Radiation Research.

## Comment from the Author

It is an unexpected pleasure and a great honor for us to receive the Terasima Award for our latest paper entitled “Estimation of β-ray dose in air and soil from Fukushima Daiichi Power Plant accident” published in JRR. The nuclear accident at the Fukushima Daiichi Nuclear Power Plant (FDNPP) occurred as a consequence of the massive earthquake and associated tsunami that struck Japan on 11 March 2011. Dose evaluations have primarily focused on γ-rays because β-rays are not directly related to effective dose. However, β-rays contribute to the 70 μm dose equivalent in the context of skin dose, and also might contribute to whole body dose for small insects. Using the Monte Carlo radiation transport code MCNP-4C, we calculated the β-ray dose for ^129m^Te, ^129^Te, ^131^I, ^132^Te, ^132^I, ^134^Cs and ^137^Cs in air as a function of altitude and in soil. These calculations of β-dose rate for each radionuclide were conducted for the conditions following the FDNPP accident, with ^137^Cs deposition assumed to be 1000 kBq/m^2^. The cumulative 70 μm β-ray dose at 30, 60 and 90 days after deposition was estimated to be 35, 45 and 53 mGy for the ground surface, and 61, 79 and 92 mGy in the soil, respectively. These results can be used to estimate the external β-ray exposure for small creatures as well as for human skin.

We are delighted that our study has been widely accepted in our research field and frequently cited.

